# Do Cellular Entry Mechanisms of SARS-Cov-2 Affect Myocardial Cells and Contribute to Cardiac Injury in COVID-19 Patients?

**DOI:** 10.3389/fphys.2021.630778

**Published:** 2021-03-09

**Authors:** Elise Balse, Stéphane N. Hatem

**Affiliations:** ^1^INSERM UMRS 1166, ICAN – Institute of CardioMetabolism and Nutrition, Sorbonne Université, Paris, France; ^2^Institut de Cardiologie, Hôpital Pitié-Salpêtrière, Paris, France

**Keywords:** SARS-CoV-2, myocardium, trafficking, COVID-19, internalization

## Abstract

Although the main vital organ affected by SARS CoV-2 is the lung, more than 20% of hospitalized patients show heart injury, however, the underlying mechanisms are still actively investigated. Inflammation or myocardial ischemia are now well-established pathogenic factors. Direct cardiac damage by the virus is likely and might account for some aspects of cardiac disease in COVID-19 patients. However, precise knowledge on mechanisms of virus entry and progression in host cells and notably in cardiac cells is necessary in order to define the broad spectrum of pathogenicity of SARS-Cov-2 on myocardium and to identify specific therapeutic targets. This review will focus on the intracellular trafficking machinery, the Achilles heel of host cells, which can be used by the virus to infect cells of the cardiovascular system.

## Review

Approximately 20% of COVID19 patients have cardiac injury (Guo et al., 2020; Shi et al., 2020) from an isolated elevation of troponin-I to a pattern of fulminant myocarditis and ventricular failure (Giustino et al., 2020; Lala et al., 2020). Rhythm disturbances, independent of hydroxychloroquine and azythomycin intake, were also reported in 16.7% of hospitalized patients and 44.4% of those in resuscitation in the Wuhan cohort.

Several mechanisms have been described explaining cardiac injury in COVID-19 patients. Firstly, inflammation of the myocardium at the acute phase or, delayed, during the cytokine storm (Paul et al., 2020) is likely the main underlying mechanism. Indeed, necrotic myocytes, infiltrates of inflammatory cells and lymphocytes compatible with early viral myocarditis has been consistently observed post mortem in the heart of COVID-19 patients (Basso et al., 2020; Fox et al., 2020). Secondly, myocardial ischemic injury due to microvascular thrombi, plaque instability and oxygen supply demand imbalance are other underlying mechanisms of cardiac injury (Giustino et al., 2020). The mode of entry and circulation of SARS-CoV-2 in the cells raises question as to the direct toxicity of the virus on myocardium also supported by the detection of SARS-CoV-2 RNA in myocardium of COVID-19 patients (Puelles et al., 2020).

Like other coronaviruses (CoVs), SARS-CoV-2 is an enveloped, positive-stranded RNA virus. The viral genome is bound to the nucleocapsid (N protein) and is enveloped by a host-derived lipid bilayer, which contains structural proteins associated with the envelop: the membrane glycoprotein (M protein), the envelope (E protein) and the spike glycoprotein (S protein) (Walls et al., 2020). Three steps are required for internalization of the viral genome: attachment to a receptor, penetration (entry), and decapsidation (release of viral content). The M and E proteins are involved in viral assembly and secretion. The S protein plays a key role in the entry of the virus into the target cell. The latter consists of two domains: S1 and S2. The S1 domain is responsible for the binding of the virus to its membrane receptor (ACE2) on the target cell, the S2 domain enables fusion of the viral and cell membranes. Cleavage of the S protein into S1 and S2 subunits (priming) and subsequent cleavage of the S2’ site by proteases is necessary to allow fusion of the virus membrane with the target cell membrane (activation) (Walls et al., 2020). It is indeed the availability of these proteases in the target cell that will determine how the virus enters the cells.

While the endocytosis pathways of SARS-CoV are not yet clearly established, coated viruses are known to use the classical host cell endocytosis pathways: clathrin vesicles (coated pits) or caveosomes. The Virus-Receptor Complex (ACE2) contained in these endocytosis vesicles then moves from one endosome to another by fusion of the membranes from a donor compartment to an acceptor compartment, with increasing acidification of the endosomal pH (pH 6.5–6.0 in the early endosome, pH 6.0–5.6 in the late endosome). The decrease in endosomal pH will trigger conformational changes in the viral glycoprotein and release of the nucleocapsid into the host cell cytoplasm (decapsidation) at the lysosome (pH 4.5–5.0).

**FIGURE 1:**
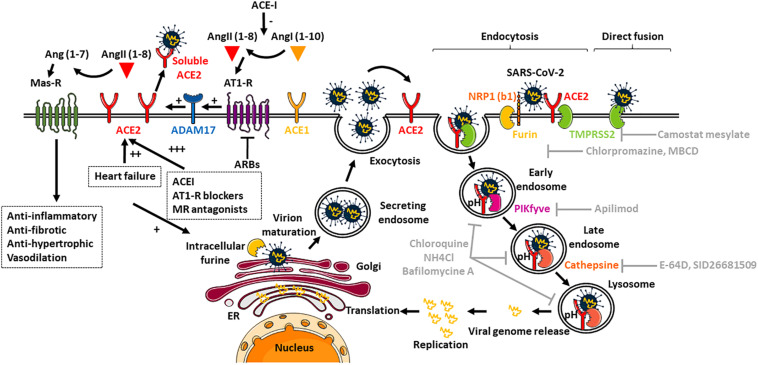
Regulation of ACE2 expression and mechanisms of entry of SARS-CoV-2. ACE2 converts AngII (1-8) to Ang (1-7), the Mas-R receptor agonist with anti-inflammatory and anti-fibrotic effects. ACE2 can also be cleaved and become plasmatic by the ADAM17 protease, which is itself activated by the AT1-R receptor. In gray lettering are indicated the different molecules currently being studied to prevent internalization of the virus and its subsequent replication in the endoplasmic reticulum (ER).

It now seems well established that SARS-CoV-2 can use the intracellular trafficking pathway of the host cell since classical lysosomotropic agents such as ammonium chloride, which increases endosomal pH, and bafilomycin A, a proton pump inhibitor, exert a strong inhibitory effect on viral load in mammalian cells *in vitro* (Hoffmann et al., 2020; Ou et al., 2020). The weak chloroquine base also acts on the endosomes by interfering with their acidification (Hu et al., 2020; Figure 1). However, direct fusion with the host membrane cannot be ruled out since recent study showed that the TMPRSS2 protease expressed by host cells is sufficient to promote virus activation and infection (Ou et al., 2021), which could explain the weak efficacy of chloroquine treatment in clinical practice. In this context, several therapeutic avenues can be considered since the stages of intracellular trafficking can be targeted: spike initiation and activation, endocytosis pathways of SARS-CoV-2 and its receptor, progression of internalization in the different endosomes and viral replication.

The endocytosis pathway currently considered is that of clathrin by inference with SARS-CoV or hCoV-NL63, although to date no studies have been performed for SARS-CoV-2 (Yang and Shen, 2020). Indeed, the use of chlorpromazine has shown that the viral load was reduced by 85% *in vitro* (Inoue et al., 2007). The use of a clathrin inhibitor, Pitstop, or a dynamin GTPase 1 and 2 inhibitor, prevents viral infection by hCoV-NL63 (Milewska et al., 2018). However, non-clathrin-dependent endocytosis pathways, such as the caveosome pathway, or non-caveolin-dependent and lipid raft-dependent pathways cannot be excluded since the use of the MCD molecule (or cyclodextrin), which destroys lipid-rich membrane microdomains, prevents viral infection to a lesser extent (30%) (Inoue et al., 2007). In this sense, another study conducted on SARS-CoV showed that MCD drastically prevents the internalization of the virus while excluding the contribution of caveolin (Wang et al., 2008).

While the involvement of different endocytosis pathways is not established for SARS-CoV-2, it should be noted that the tropism of the virus and its infectivity will depend on the host cell’s endocytosis machinery, the possibility of using different pathways depending on the cell type, and most likely on the lipid composition of the membrane. If, at this stage, the mechanisms of entry in the different myocardial cell types (e.g., fibroblasts, inflammatory cells, vascular endothelium, etc.) is not clear, one should note that both caveolin-dependent and clathrin-dependent endocystosis pathways co-exist, for instance, in cardiac myocytes (Melgari et al., 2020). In any case, published studies on SARS-CoV or hCoV-NL63/ACE2 show the rapidity of the internalization process since SARS-CoV/ACE2 or hCoV-NL63/ACE2 complexes are localized in early endosomes a few minutes to an hour after infection (Inoue et al., 2007; Wang et al., 2008; Milewska et al., 2018).

The priming and activation of the spike protein required for fusion of the viral envelope to the host cell membrane is dependent on proteases. Depending on the viral strain and the host cell, one or more host proteases may be involved in the cleavage of the S-protein, such as furin, trypsin, cathepsins, TMPRSS-2 and -4 (transmembrane serine protease) and HAT airway trypsin-like protease (HAT). Among the studies performed on SARS-CoV-2, two major families of proteases appear to be involved in activation: serine proteases (membrane localization) and cysteine proteases (endo/lysosomal localization). Early studies performed on SARS-CoV-2 agree to show the major role of serine proteins such as TMPRSS or furin (Hoffmann et al., 2020; Ou et al., 2020). Due to their membrane localization, these serine proteases would be internalized at the same time as the SARS-CoV-2/ACE2 complex and would intervene in the fusion processes with early endosomes (Hoffmann et al., 2020; Ou et al., 2020). The use of camostat mesilate, a serine protease inhibitor already clinically approved in Japan for other applications, drastically inhibits viral load: 50% in cells expressing TMPRSS2 (Hoffmann et al., 2020). Cysteine proteases, notably cathepsin L, would take over to allow the complex to fuse at the lysosome membrane level. For example, specific inhibition of cathepsin L with SID26681509 reduces viral load by 75% in different cell types *in vitro* (Ou et al., 2020). Similarly, in cells that do not express TMPRSS2, inhibition of cathepsins alone results in a complete reduction of viral load (Hoffmann et al., 2020). Conversely, cells expressing TMPRSS2 are less sensitive to the cathepsin inhibitor and viral load can be fully reduced if camostat mesilate is used in combination with a cathepsin B/L inhibitor such as E-64D (Hoffmann et al., 2020). Thus, these different proteases will play more or less determining roles depending on the cell type considered, which may also explain the tropism of the virus and its infectivity. It is important to note that any exogenous protease (trypsin, papain) could promote the entry of the virus into cells (Letko et al., 2020). Finally, the existence of a furin site between S1 and S2 on the spike protein could also promote the pathogenicity of SARS-CoV-2 (Ou et al., 2020; Walls et al., 2020). It is interesting to recall that the activity of the serine protease furin is increased in patients with heart failure (it cleaves proBNP into active BNP) and is highly expressed in T lymphocytes (Ichiki and Burnett, 2014).

The progression of internalization in the different endosomes is regulated by a large number of molecular actors: the endosomes themselves, associated with small Rab GTP-ases proteins, the molecular motors that allow the movement of these endosomes along the cytoskeleton (actin microtubules and microfilaments), and the SNAREs proteins that allow fusion from one endosomal compartment to the other up to the lysosome where the virus will be decapsidated. In this context, the study by Ou and colleagues suggests the importance of PIKfyve, an enzyme in the synthesis of phosphatidylinositol-3,5-bisphosphate (PI(3,5)P_2_) in the early endosome. PI(3,5)P_2_ regulates the maturation dynamics of the early endosome into the late endosome. *In vitro*, the use of Apilimod, a PIKfyve inhibitor, significantly decreases the viral load induced by SARS-CoV-2 (Ou et al., 2020). Finally, the inhibition of TCP2, a PI(3,5)P_2_ target in the lysosome, by Tetrandrin effectively blocks the entry of the virus (Ou et al., 2020).

Using a single nuclei RNA sequencing approach allowing identification of all the cells within a tissue by their transcriptomic profile, then grouping them into sub-populations (gene cluster), mapping of ACE2 expression in the myocardium has been achieved. This work revealed that cardiomyocytes, vascular wall cells (pericytes, smooth muscle cells), and fibroblasts strongly express ACE2 (Nicin et al., 2020). From these RNAseq studies, further analysis of the various entry pathways for SARS-CoV-2 is now required. In addition, the functionality of these different pathways and the kinetics of virus entry have to be investigated in primary cell culture models.

Other mechanisms could promote the impact of SARS-CoV-2 on the heart. These include the Notch signaling pathway that regulates homeostasis of the cardiovascular system during ontogenic development, atherosclerosis or post-ischemic myocardial remodeling. Notch positively regulates furin and negatively regulates ADAM17, the latter favoring ACE2 shedding. Two recent studies pointed the role of the membrane protein neuropilin-1 (NRP1) in increasing virus uptake in different cell types, including respiratory and olfactory epithelial cells which express low levels of ACE2 (Cantuti-Castelvetri et al., 2020; Daly et al., 2020). Proteolytic cleavage of the of the spike protein by furin release a polybasic Arg-Arg-Ala-Arg carboxyl-terminal sequence on S1 that favors interaction with NRP1. This interaction does not modify virus binding but accelerate cellular entry of SARS-CoV-2 in cell cultures and in the olfactory epithelium. Of note, NRP1 is highly expressed in the cardiovascular system (Wang et al., 2015), notably in endothelial cells, vascular smooth muscle cells and cardiomyocytes, and is upregulated during epicardial activation (Lowe et al., 2019). Thus, prevention of Notch activation could represent a strategy to interfere with the virus entry into the cells by reducing furin activity.

## Concluding Remarks

Although recent publications support the role of ACE2 in SARS-CoV-2 infection of cardiomyocytes (Bojkova et al., 2020; Liu et al., 2020), cardiac cells appear to be protect against TMPRRSS2-mediated virus entry (Liu et al., 2020). However, other proteases such as cathepsins, that can cleave the spike protein, could have a predominant role in this organ (Bojkova et al., 2020).

The diversity of internalization pathways of the virus that can coexist in the same cell type and may substitute for each other as well as the various proteases that can prime the virus and compensate for each other could determine the tropism and infectivity of the virus. Furthermore, various clinical conditions are associated with the dysregulation of trafficking machinery of cardiomyocytes including changes in cellular cholesterol content and lipid composition of the membrane and endosomes which affects intracellular trafficking or excessive shear stress of the plasma membrane that stimulate cell adhesion receptors (Balse et al., 2009; Boycott et al., 2013). Therefore, the initial stress state of the cardiac cells could be a major determinant of their infectivity by the virus and contribute to the vulnerability to the infection of patients with cardiopathies.

## Author Contributions

EB and SH wrote the manuscript. Both authors contributed to the article and approved the submitted version.

## Conflict of Interest

The authors declare that the research was conducted in the absence of any commercial or financial relationships that could be construed as a potential conflict of interest.
